# Supplementary material for: Mechanisms of Trichomes and Terpene Compounds in Indigenous and Commercial Thai Rice Varieties against Brown Planthopper
Source: Insects. 2022 May 1;13(5):427. doi: 10.3390/insects13050427 (PMC9143670; doi:10.3390/insects13050427)
Supplement: Supplementary file 1 [file insects-13-00427-s001.zip › insects-1635179-supplementary.pdf]

Supplementary Table S1. Volatile profiles from rice test of Terpene and Derivatives and Relative Abundance (%) by constitutive and inducible release methods.

Rice varieties	Compounds	% Relative Abundance	
		Constitutive defense	Induced defense
RH	Cyclobutane, 1,3-diisopropenyl-, trans	2.098	6.958
	1,5-Heptadiene, 3-methyl-, (E)-	0.063	-
	4'-Ethylpropiophenone	0.17	-
	Ethanone, 1-(4-ethylphenyl)-	0.606	-
	α-Cubebene	3.366	-
	Naphthalene, 1,2,3,4,4a,-7 hexahydro-1,-6dimethyl--1)-4 methylethyl)-	3.366	-
	1-Octene, 2-methyl-	0.079	-
	1-Hepten-3-one	0.463	-
	4-Undecene, 6-methyl-	0.463	-
	Decane, 3,8-dimethyl-	0.319	-
	Tridecane, 2,2,4,10,12,12-hexamethyl-7-(3,5,5-trimethylhexyl)-	0.319	-
	Cyclopropane, 1-(2-methylbutyl)-1-(1-methylpropyl)-	7.86	-
	β-Curcumene	1.404	-
	Hexane, 3-methyl-4-methylene-	0.377	-
	3-Undecene, 5-methyl-	0.217	-
	3-Ethyl-3-hexene	0.107	-
	5-Hepten-3-one, 5-ethyl-4-methyl-	0.102	-
	cis-Muurola-4(15),5-diene	-	2.671
	β-Sesquiphellandrene	-	6.758
Suphan Buri 1	α-Phellandrene	0.067	-
	D-Limonene	11.616	-
	Naphthalene	10.386	2.046
	α-Cubebene	7.027	-

Rice varieties	Compounds	% Relative Abundance	
		Constitutive defense	Induced defense
	5-Hepten-3-one, 5-ethyl-4-methyl-	0.159	-
	Hexane, 3,4-bis(1,1-dimethylethyl)-2,2,5,5-tetramethyl-	0.15	-
Hawm Dawk Doo	Benzene, (2,2-dimethylpropyl)-	2.155	-
	Benzene, [(cyclohex-1-en-1-yl)methyl]-	1.873	-
Gled Plah Chawn	Cyclobutane, 1,3-diisopropenyl-, trans	9.659	18.95
	Naphthalene	6.04	6.062
	3-Pentanone, 2,2,4,4-tetramethyl-	0.23	-
	Hexane, 3,4-bis(1,1-dimethylethyl)-2,2,5,5-tetramethyl-	0.07	-
	Naphthalene, 1,2,3,4,4a,7-hexahydro-1,6-dimethyl-4-(1-methylethyl)-	3.855	-
	Bicyclo[5.2.0]nonane, 2-methylene-4,8,8-trimethyl-4-vinyl-	4.219	-
	1,3,4,6-Hexanetetrone, 1-(4-methylphenyl)-6-phenyl-	0.291	-
	1,3-Cyclohexadiene, 5,6-dimethyl-	0.101	-
	1-Methyl-3-(1'-methylcyclopropyl)cyclopentene	0.297	-
	Santolina triene	0.853	-
	β-Myrcene	1.658	0.171
	Cyclohexene, 1-(1-propynyl)-	0.14	-
	Caryophyllene	-	27.66
	Geijerene	-	1.686
Sahm Ruang	Benzene, 1,2-diethyl-	36.204	-
	Benzene, 1,4-diethyl-	37.083	-
	Cyclopentanone, 2,2,5-trimethyl-	0.074	-

Rice varieties	Compounds	% Relative Abundance	
		Constitutive defense	Induced defense
	α-Cubebene	-	7.005
	2,5-Cyclohexadien-1-one, 4,4'-(1,2-ethanediylidene)bis[2,6-bis(1,1-dimethylethyl)-	-	0.024
	Cyclobutanone, 2,3,3,4-tetramethyl-	-	0.892
Kam Pai	Cyclobutane, 1,3-diisopropenyl-, trans	60.606	-
	Tridecane, 2,2,4,10,12,12-hexamethyl-7-(3,5,5-trimethylhexyl)-	1.376	-
	Octadecane, 2,2,4,15,17,17-hexamethyl-7,12-bis(3,5,5-trimethylhexyl)-	1.368	-
	Hexane, 3,4-bis(1,1-dimethylethyl)-2,2,5,5-tetramethyl-	0.029	-
	1,5-Heptadiene, (Z)-	0.862	-
	Aromandendrene	3.614	-
Khao Maew	Cyclobutane, 1,3-diisopropenyl-, trans	12.799	5.207
	Benzene, 1,4-diethyl-	0.107	34.845
	Naphthalene	0.032	5.536
	Caryophyllene	12.567	-
	4'-Ethylpropiophenone	-	0.443
	(S,1Z,6Z)-8-Isopropyl-1-methyl-5-methylenecyclodeca-1,6-diene		3.072
KDML105	Cyclobutane, 1,3-diisopropenyl-, trans	12.216	-
	Naphthalene	0.039	1.638
	cis-Muurola-4(15),5-diene	5.596	-
TN1	1,3-Bis(cyclopentyl)-1-cyclopentanone	0.066	-
	Bicyclo[3.1.0]hex-2-ene, 4-methyl-1-(1-methylethyl)-	0.055	-
	α-Phellandrene	0.847	-

Rice varieties	Compounds	% Relative Abundance	
		Constitutive defense	Induced defense
	Cyclobutane, 1,2-bis(1-methylethenyl)-, trans-	10.351	-
	Hexane, 3,4-bis(1,1-dimethylethyl)-2,2,5,5-tetramethyl	0.162	-
	Naphthalene	10.087	6.154
	1H-Indene, 1-hexadecyl-2,3-dihydro-	0.125	-
	β-Caryophyllen	23.253	-
	1,5-Heptadiene, (E)-	-	0.057
	Ethanone, 1-(4-ethylphenyl)-	-	2.179
	1-Penten-3-one	-	0.079
Beu Sim	Cyclobutane, 1,3-diisopropenyl-, trans	12.799	-
	4-(2', 4', 4'-trimethyl-yciclo[4.1.0]hept-2'-en-3'-yl)-3-buten-2-one	12.567	-
	Cyclobutane, 1,2-dipropenyl-	-	8.811
	Benzene, 1,3-diethyl	-	30.096
	Benzene, 1,4-diethyl-	-	30.751

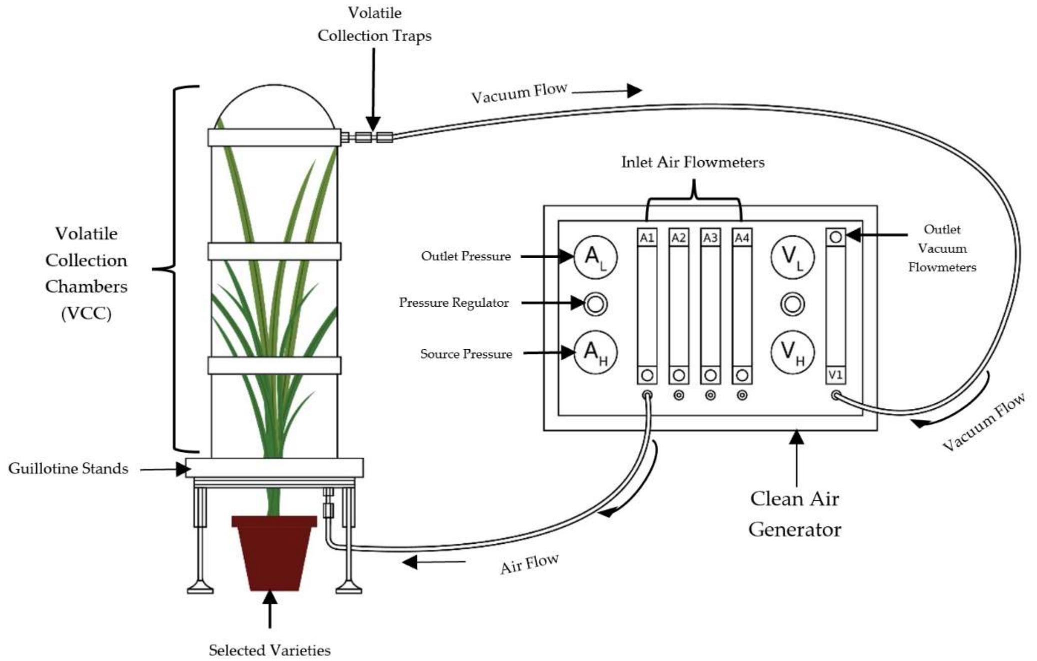

Supplementary Figure S1. Volatile collecting trap system using potted plants with and without BPH infestation.

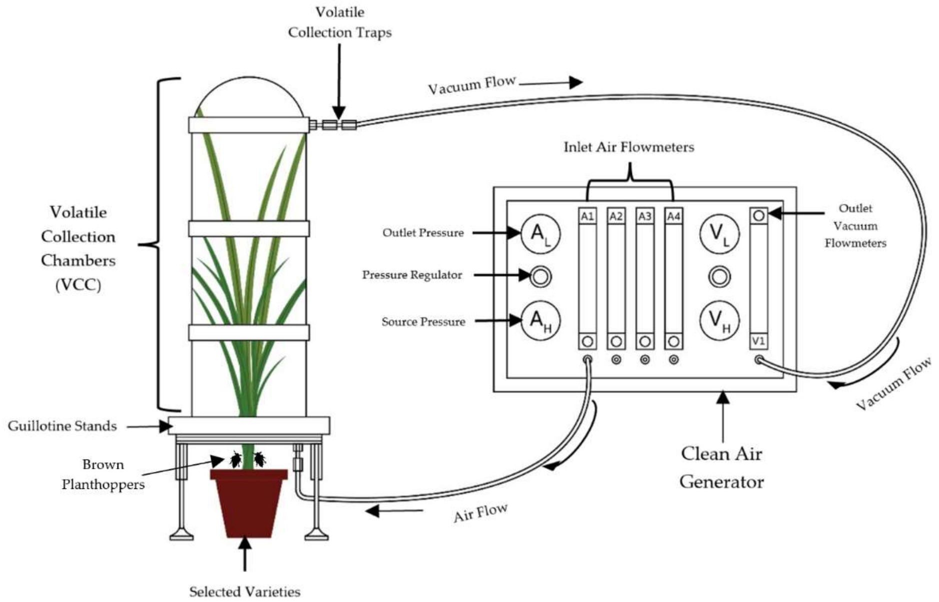

Supplementary Figure S2. Volatile collecting trap system using potted plants with BPH infestation.

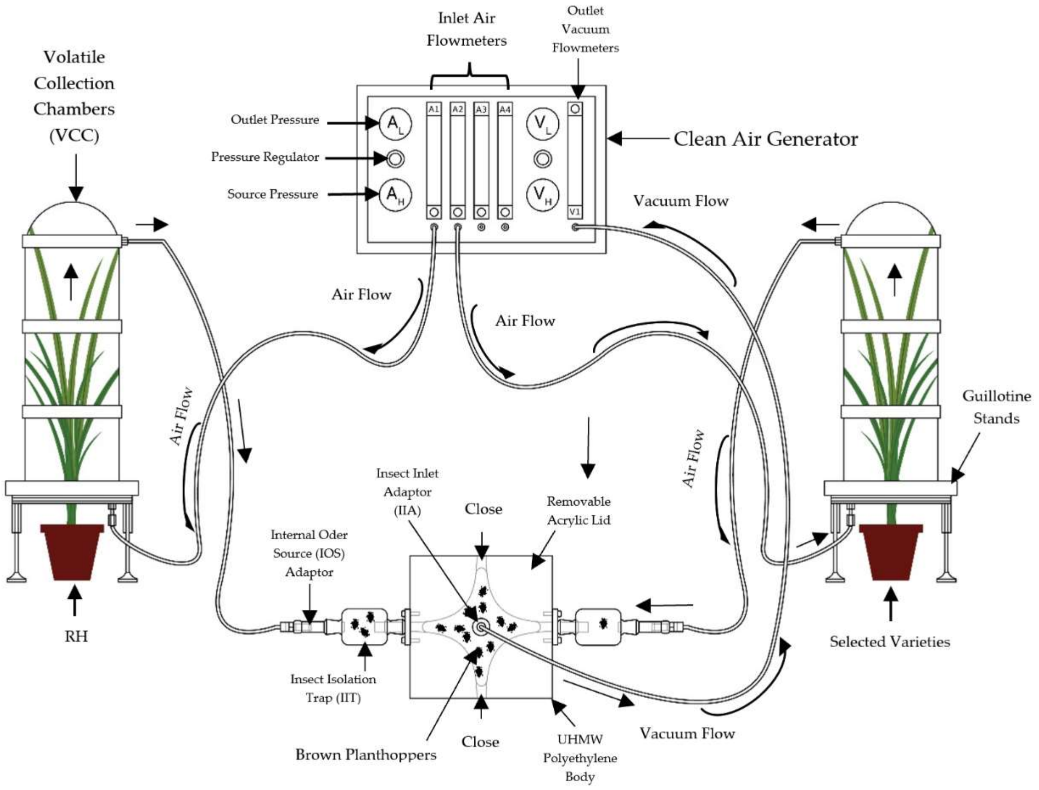

Supplementary Figure S3. Two-choice arena olfactometer system test using potted plants without BPH infestation.

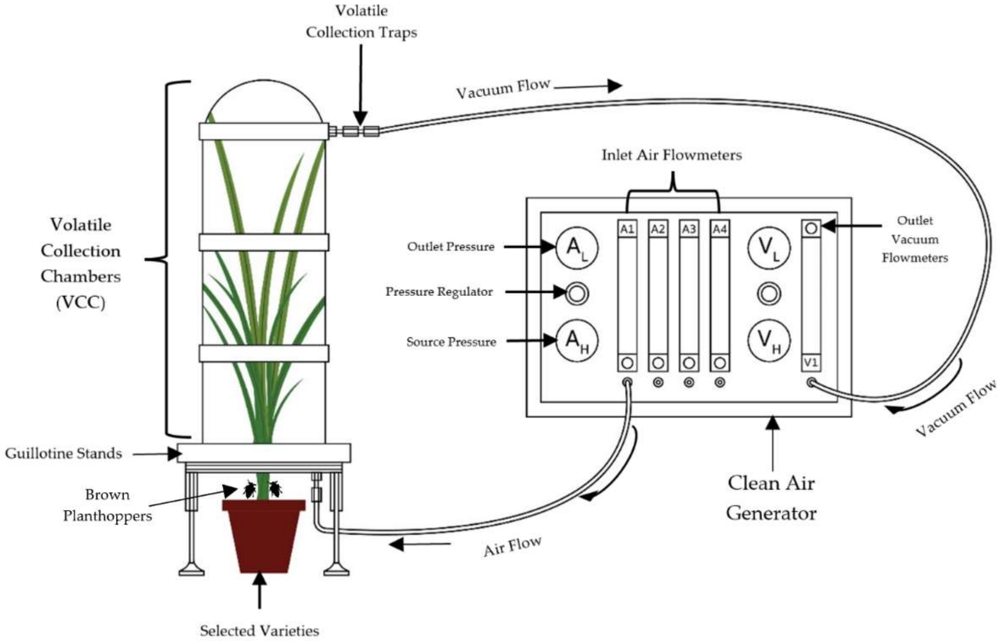

Supplementary Figure S4. Two-choice arena olfactometer system test using potted plants with BPH infestation.
